# Family planning need of people living with HIV/AIDS in antiretroviral therapy clinics of Horro Guduru Wollega zone, Ethiopia

**DOI:** 10.1186/s13104-017-2914-0

**Published:** 2017-11-09

**Authors:** Reta Tsegaye

**Affiliations:** Department of Nursing and Midwifery, College of Health Sciences, Wollega University, P.O. Box 395, Nekemte, Ethiopia

**Keywords:** Family planning, People living with HIV (PLWH), Horro Guduru Wollega, Mother to child transmission

## Abstract

**Objective:**

The purpose of this study was to identify factors associated with family planning needs among people living with human immunodeficiency virus (HIV) in Ethiopia.

**Results:**

Three hundred twenty-one participants provided information on family planning methods and associated factors. Forty-six-point four percent of respondents reported using at least one form of family planning method; injectables (50.3%) and condoms (70.2%) were the most commonly used type of family planning method before and after HIV diagnosis, respectively. Age, the desire to have children, and the desire to have more than two children were significantly associated with the use of family planning methods.

**Electronic supplementary material:**

The online version of this article (10.1186/s13104-017-2914-0) contains supplementary material, which is available to authorized users.

## Introduction

By the end of 2015, the number of people living with HIV had reached an estimated 33.3 million. Women account for half of the estimated adults living with HIV and AIDS worldwide, the majority of whom are in their reproductive years [1, 2]. With increased access to treatment for PLHIV, there has been a consequent decrease in mortality among PLHIV and subsequent increase in normal functioning including sexual activity [3].

Many women are at risk for unintended pregnancy and HIV infection at the same time. In a number of African countries the rate of unintended pregnancy among women living with HIV range from 51 to 84% [4]. Family planning has a major impact on improving the overall health of a woman as well as that of her children by delaying first births, reducing the total number of children born to a woman, and preventing unintended pregnancies. Nearly one-third of maternal deaths could be prevented by meeting unmet need for family planning [5, 6].

Despite these benefits, in sub Saharan Africa, family planning among PLWH is not widely used [4]. A national survey conducted in Malawi among HIV positive women revealed that 51.2% of the respondents were using family planning methods and unmet need of FP methods were 21.9% [7]. According to a cross-sectional survey conducted in Tigray region, Ethiopia, 44.3% of the respondents were using contraceptive methods at time of survey [8]. A cross sectional study conducted in West Shewa zone, Ethiopia also revealed that less than half (43.1%) of study participants were using family planning during the study period [9].

Factors associated with low utilization of family planning among PLWH vary across the countries and study sites. In the study area there is no available evidence on the use of family planning methods among PLWH. Thus, this study was done to explore factors associated with family planning needs among PLWH in Horro Guduru Wollega zone, Ethiopia.

## Main text

### Methods

#### Study site

The study was conducted in Horro Guduru Wollega (HGW) Zone, Oromia National Regional State. HGW is one of twenty zones of the region which has ten districts with different climatic conditions. This zone has a total population of 570,040 (285,515 male and 284,525 female) of which 11.36% of the population were urban inhabitants [10]. Shambu is the capital town of the zone and found 310 km from country’s capital, Addis Ababa.

#### Study population and design

The study involved cross sectional institution based study among randomly selected PLWHA who were in reproductive age and had taken at least one visit at antiretroviral therapy (ART) care from the selected ART care unit from March 31 to April 30, 2012. There were ten health care institutions (one hospital and nine health centers) providing ART services in HGW Zone. Among these, one hospital and two health centers were randomly selected. The source population was all PLWH (men and women) following ART care unit in selected HGW zone ART care units during the study period. Study populations were randomly selected who were in reproductive age and had taken at least one visit at ART care from the selected ART care unit during the study period. Those who were critically ill and could not provide informed consent were excluded from the study.

#### Sample size determination and sampling technique

The sample size was determined using single population proportion formula taking the proportion of HIV positive individuals who received ART treatment and had the desire to have children is 40% from study done in Addis Ababa [11], 5% marginal error, 95% confidence interval. A total of 1050 eligible participants were identified. Since the total number of people living with HIV/AIDS who were following ART clinics in HGW zone in ten ART clinics was < 10,000, correction formula was used. To compensate the non-response rate, 10% of the determined sample was added up on the calculated sample size and the final sample size was 323. Then the study participants were selected by systematic random sampling of every 3rd person from three study sites proportional their size. The dependent variable was family planning need, and the independent variables were socio-demography, fertility desire, and number of children desired.

#### Data collection methods and tools

Data was collected using structured questionnaire with closed and open ended questions. The questionnaire was adapted from different literatures [9, 11] (Additional file 1). The questionnaires were prepared in English, translated into Afan Oromo (working language of Oromia region), and then retranslated back to English by people who are proficient in both languages to maintain the consistency of the questionnaires. The questionnaire contained questions on socio-demographic characteristics, information about family planning use, choice and demand, and sexual behavior and condom use. Six data collectors were selected from the study site. Training was given for 2 days about the objective and relevance of the study, confidentiality of information, respondents’ rights, informed consent and techniques of interview. Three supervisors were also selected from each site and closely followed the data collection process with principal investigator. Field questionnaires were reviewed each night and any issues encountered during data collection procedures were addressed accordingly.

#### Data processing and analysis

The data cleaning was done, entered into a computer by using EPI Info version 6.5. The data were then exported to SPSS windows version 20.0 for further analysis. The descriptive analyses such as proportions, percentages, frequency distribution and measures of central tendency were conducted. Bivariate analysis of demographic variables and family planning need was described. Odds ratio was used to check significant association between dependent and independent variables. Then all variables found to be significant at bivariate level (p value < 0.05) were entered into multivariate analysis using the logistic regression model to test the significance of the association.

### Results

#### Socio demographic characteristics of study participants

Three hundred twenty-one respondents participated in the study among 323 eligible clients in the selected ARV care units, giving a response rate of 99.4%. The majority 156 (48.6%) of the respondents were between 30 and 39 years of age. The mean age (SD) of females and males were 31.4 (± 5.8) and 35.6 (± 7.9) respectively. The age range of the respondents was from 19 to 58 years. The majority more than half of the respondents were orthodox (51.4%) in religion. Nearly one-third (29.65) of the study participants can’t read and write (see Table 1).

**Table 1:** Socio demographic characteristics of respondents attending ART treatment units, Horro Guduru Wollega zone, Ethiopia, 2012

Characteristics	Frequency	Percentage (%)
Age (years)		
18–29	106	33
30–39	156	48.6
≥ 40	59	18.4
Sex		
Female	195	60.7
Male	126	39.3
Religion		
Orthodox	165	51.4
Protestant	136	42.4
Muslim	13	4
Others	7	2.2
Educational status		
Can’t read and write	95	29.6
Read and write	100	31.2
Primary	57	17.7
Secondary	52	16.2
Post secondary	17	5.1
Ethnicity		
Oromo	272	84.7
Amhara	45	14
Others^a^	4	1.3
Marital status		
Married	186	57.9
Single/never married	57	17.8
Widowed	40	12.5
Divorced	38	11.8
Occupation		
House wife	135	42.1
Farmer	51	15.8
Daily laborers	46	14.3
Government/private employee	33	10.3
Jobless	33	10.3
Merchants	23	7.2
Income		
No regular income	126	39.3
≤ 445	147	46.0
> 445	48	14.7

^a^Guraghe, Tigre

#### Family planning need, choice and use

One hundred forty-nine (46.4%) were using different types of family planning methods during the survey period. Among these 120 (80.5%) and 50 (33.6%) were using condom and injectables respectively. Out of those who were not using family planning method at the time of survey period, only 58 (33.7%) of them have intended to use family planning methods in the future (Table 2).

**Table 2:** Distributions of study participants by current use and future need of family planning methods in Horro Guduru Wollega zone, Ethiopia, 2012

Contraceptive use n (%)	Current use n (%)	Future need to use n (%)^a^
Yes	149 (46.4)	58 (33.7)
No	172 (53.6)	114 (66.3)

Contraceptive methods	Current^b^	Future^c^
Injectables	50 (33.6)	18 (31)
Condom	120 (80.5)	31 (53.4)
OCPs	5 (3.3)	4 (6.9)
Implants	9 (6)	9 (15.5)
Others	1 (0.7)	1 (1.7)

^a^Among current contraceptive non users (n = 172)

^b^Among current contraceptive users (n = 149) and more than one FP methods were reported use

^c^Among those who want to use contraceptive in the future (n = 58) and more than one FP methods were reported need in the future

#### Sexual behavior and condom use

One hundred ninety-six (61.1%) of the study participants were sexually active within the past 6 months. Among these 128 (65.3%) reported using condoms while 68 (34.7%) did not use. Out of those who reported condom use, the majority 115 (89.8%) used it regularly. The most common reason for condom use was advice from health professionals 57 (44.5%) while wanting to have child 39 (57.3%) was the most common reason for non condom use.

#### Factors associated with family planning need

From multivariate analysis, those ages group 18–29 were more likely want to use family planning methods than those above thirties (AOR: 2.5, 95% CI 1.18–5.46). Study subjects who desired children were more likely to want to use family planning in the future than those who did not desire children (adjusted OR: 2.8, 95% CI 1.2–6.6). On the other hand those who desired to have less than two children were less likely to use any family planning in the future than their counter parts (adjusted OR: 0.26, 95% CI 0.1–0.8) (Table 3).

**Table 3:** Association of future family planning need by selected variables among PLWH following ART unit, Horro Guduru Wollega zone, Ethiopia, 2012

Characteristics	Need for future FP N (%)	Do not need for future FP N (%)	Crude OR (95% CI)	Adjusted OR (95% CI)
Age				
18–29	26 (55.3)	27 (28.4)	*3.1 (1.5, 6.5)*	*2.5 (1.18, 5.46)*
30–39	21 (44.7)	68 (71.6)	1	1
Sex				
Male	25 (43.9)	33 (28.2)	2 (1.03, 3.89)	1.6 (0.7, 3.8)
Female	32 (56.1)	84 (71.8)	1	1
Marital status				
Married	25 (43.9)	40 (34.2)	1.5 (0.8, 2.9)	–
Single/divorced/widowed	32 (56.1)	77 (65.8)	1	1
Educational status				
Illiterate/read and write	38 (66.7)	80 (68.4)	0.9 (0.5, 1.8)	–
Primary and above	19 (33.3)	37 (31.6)	1	
Future child desire				
Yes	41 (71.9)	46 (39.3)	*4 (2.0, 7.9)*	*2.8 (1.2, 6.6)*
No	16 (28.1)	71 (60.7)	1	1
Number of children desired				
≥ 2	32 (78)	23 (50)	*3.6 (1.4, 9.1)*	*3.8 (1.25, 11.7)*a
< 2	9 (22)	23 (50)	1	1

Italic shows significant association at p < 0.05

### Discussion

The current study was undertaken to describe family planning need among people living with HIV/AIDS in Horro Guduru Wollega zone, Ethiopia. In this study about 44.5% of the respondents had used at least one method of family planning methods before HIV diagnosis and this number was increased to 57.6% after HIV diagnosis. This finding was similar with a study conducted among HIV positive women in South Gondar and North Wollo zone, Ethiopia and South Africa [12, 13]. The reason for increased utilization of family planning may be attributed to the counseling services they got from their ART providers to prevent unintended pregnancies and prevention of HIV re-infection from their partners. Among those who were not currently using, only 33.7% did want to use family planning in the future; indicating that counseling for family planning for these people still needs to be addressed.

Sixty-one point one percent of the study participants were sexually active 6 months preceding the survey. This finding was higher than study conducted in Bahir Dar city (50.4%) of Ethiopia but lower compared to study conducted among serodiscordants in Henan, China (83.6%) [14, 15]. The increase in sexual activity among PLWH could be attributed to improved quality of life because of antiretroviral therapy, opportunistic infection prophylaxis and other health care packages [3]. With increased access to ART made possible by global and national initiatives, more HIV positive people are increasingly becoming healthier and more sexually active [3, 16].

Of those who were sexually active, the majority 128 (65.3%) reported using condom while 68 (34.7%) never used at all and among who reported using condoms, over 10% inconsistently used them. These findings were similar to study conducted in Malawi (65.8%) and Nepal (65.8%) reported condom use but lower compared to study conducted in Uganda (77.6%) [17–19]. The results appear to indicate significant proportions of PLWH were increasingly practicing risky sexual behavior which has a greater risk of horizontal HIV transmission. So counseling regarding the importance of condom usage in conjunction with more effective modern contraceptive methods must continue to be reinforced over the course of ongoing ART treatment to prevent both infection and pregnancy. Oral pre-exposure prophylaxis (PrEP) for heterosexual HIV discordant couples has proven feasible to reduce HIV transmission and this may be considered as possible additional intervention for the uninfected partner [20].

This study identified many factors like age; desire to have children and number of children desired as important factors associated with future family planning need. Those who were age group between 18 and 29 were more likely to use family planning than those above thirties which was similar to study conducted in Uganda and Tigray region [21, 22]. This may be attributed to the possible need of these populations to prevent unintended pregnancy, and delay to have children as their age is comparably younger than their counter parts. However, according to study conducted in Addis Ababa, age has no significant association with future family planning need.

The other factor associated with future family planning use was future desire for children. Those who desired children were three times more likely to use future family planning than those who did not desire children. Similarly study conducted in Addis Ababa, North Shewa (unpublished) and West Shewa zones of Ethiopia identified that those who desired children were more likely to use family planning in the future than their counter parts [9, 11]. This may be again the possible need of these populations to limit and space the number of children. But according to study conducted in Kenyan health facility, those expressed fertility desire were less likely to use family planning in the future [23].

Number of children desired was another factor for future family planning need identified in this study; those who desired two and above children were more likely to use family planning methods than those who desired one. This may be again due to the possible desire of these populations to limit and space births as their desired number of children increases. This finding was comparable with the study conducted in slums of Nairobi, Tigray region and Debre Markos of Ethiopia [8, 24, 25] as respondents who have one or more living children were more likely to use contraceptive compared with those with no child.

## Limitation of the study

- The cross-sectional nature of the data could not allow the causal effect relationships of dependent and independent factors.
- In this study, sexually active refers to sexual relationship between male and female only. Men sex with men (MSM) was not included as it is culturally inappropriate in the study area.
- Even though data collectors were trained on respondent’s confidentiality, respondents may still provide desired answers by data collectors especially on sexual behaviors. Thus, social desirability bias may not be totally avoidable.
- Despite these limitations, the data being gathered possess a better description of family planning need of PLWH in the study area.

## Additional file

**Additional file 1.** Questionnaire. Information sheets, Consent form and questionnaire used during the study.

## Abbreviations

AIDS: acquired immune deficiency syndrome
ART: antiretroviral therapy
HGW: Horro Guduru Wollega
HIV: human immunodeficiency virus
PLWH: people living with HIV
SPSS: Statistical Packages for the Social Sciences
