# Supplementary material for: Family planning need of people living with HIV/AIDS in antiretroviral therapy clinics of Horro Guduru Wollega zone, Ethiopia
Source: BMC Res Notes. 2017 Nov 9;10:581. doi: 10.1186/s13104-017-2914-0 (PMC5679377; doi:10.1186/s13104-017-2914-0)
Supplement: Supplementary file 1 — Additional file 1. Questionnaire. Information sheets, Consent form and questionnaire used during the study. [file 13104_2017_2914_MOESM1_ESM.docx]

## Subject information sheet

**Addis Ababa University**

**College of Health Sciences**

**Department of Nursing and Midwifery**

Here, I the undersigned, at Addis Ababa University College of Health Sciences department of nursing and midwifery, currently undertaking research on a topic entitled Fertility desire and family planning need among People Living with HIV/AIDS (PLWHA) in selected ART clinics of Horro Guduru Wollega Zone, North West Oromia, Ethiopia

For this study, you were selected as a participant. And before getting your consent or permission of your participation, you need to know all necessary information related to the study. Thus, this information will be detailed as;

**Objective**: To assess fertility desire and family planning need among PLWHA following ARV care unit in selected ART clinics of Horro Guduru Wollega Zone, Oromia National Regional State

**Significance of the study**: In our country, there is only limited information available about fertility desire of women and men living with HIV/AIDS. Some women may have children even after HIV diagnosis and very little is known about whether the pregnancies were intentional or not.

In addition, the majority of HIV positive men and women are of reproductive age and major mode of transmission in Ethiopia is heterosexual and vertical transmission. (12) Therefore a better understanding of fertility desire and family planning need of HIV positive people is important since ARV medication is becoming available and the study is also intended to identify some of the factors influencing their fertility desire and family planning need which might help in the prevention, control and policy formulation on the preventive strategy of HIV/AIDS

- **Participants to be included**: All people living with HIV/AIDS following ART care units who have at least visited once
- **Confidentiality:** All information you give will be kept confidential and won't be accessible to any third party. Your name won't be registered on the question sheet so that you will not be identified.
- **Risks and Benefits of the study**

**Risks:** the study has no clear and identifiable risks except perceived fear of respondents

**Benefits:** For your participation in the study no payment will be granted or has no any special privilege to you. But, participating in the study and giving your information to questions asked will have great input in efforts of prevention of transmission of HIV/AIDS

- **Consent:** Your participation in the study will be totally based on your willingness. You have the right not to participate from the beginning, or stop any time after starting participation. You will not be forced to respond to the information you do not know

**II. Consent Form**

My name is -----------. We are working with the research team of Addis Ababa University. Here at -------Hospital/Health center ARV treatment unit we are interviewing men and women PLWHA on follow up care to evaluate their need for family planning .We believe that this study would help to bring change in fertility and family planning services for HIV positive people on ARV treatment. We would like to assure you your name will not be mentioned in the questionnaire and the information that you will give us will be kept confidential and only used for research purpose. You have full right to refuse to take part or to interrupt the interview at any time. But the information that you will give us is quite useful to achieve the objective of the study and to bring change in the fertility and family planning service provision for HIV positive people on ARV treatment

Are you willing to participate in the study?

1- Yes 2 - No

If the answer is yes, thanks! Conduct the interview.

If the answer is no, Thanks!

Don’t force or reinforce an individual to participate in the survey

Interviewer’s code ---------name ------------------ signature -------

Date of interview ----------- date ---------------month

Supervisor’s name ----------------signature --------------year

Checked on ----------- date---------------month------year

Complete 1

Incomplete 2

Other (specify) -----

Principal Investigator- Reta Tsegaye

Address – email- [retatg@gmail.com](mailto:retatg@gmail.com)

**PART I – Socio - demographic characteristics**

| No | Questions | Categories |
| --- | --- | --- |
| 101 | How old are you? | ------ Years (age in completed years) |
| 102 | What is your Sex? | Male -------- 1  Female -------- 2 |
| 103 | What is your religion? | Orthodox ------- 1 Catholic ---- 2  Muslim ------ 3 Protestant -- 4  Others (specify) ---------- 89 |
| 104 | What is the highest Educational level you  completed? | ---------- Grade completed  Able to read and Write ------------ 86  Un able to read & Wright ------------ 87  No response ------------99  Other specify ---------------89 |
| 105 | What Ethnic group do you belong to? | Oromo ------------ 1 Amhara ------2  Gurage ------------ 3  Other (Specify) ------------89 |
| 106 | What is your Current Marital / relationship status? | Married------------ 1 Single -----2  Widowed ------------ 3 Divorced ----4  Non married partner --------- 5  No response ------------99 |
| 107 | What is your total Monthly income? | --------- Eth.Birr  No income ------------ 1  Don’t know ------------ 2  No response ------------ 99  Other (specify)-------------89 |
| 108 | What is your current Occupation? | Unemployed ------------ 1  Student ------------ 2  House wife ------------ 3  House servant ------------ 4  Daily laborer ------------ 5  Merchant ------------ 6  Sex worker ------------ 7  Government employ ------------ 8  Private employ ------------ 9  Other (specify)------------------ 89 |

**PART II –Information on contraceptive use, demand and choice**

| 109 | Have you (your partner) ever used family planning method before HIV diagnosis? | Yes ----------- 1  No ----------- 2  Don’t remember --------- 3  Don’t know --------- 4  No response ----------- 99  Other specify ----------89 |
| --- | --- | --- |
| 110 | If yes for Q116 specify the method you /your partner used?  (More than one answer can be possible) | Abstained from sex --------- 1  Condom ----------- 2  Pill (OCP) ----------- 3  Injectable ------------ 4  IUD ---------5-  Implant-----------------6 |
| 111 | Have you (your partner) ever used family  planning method after HIV diagnosis? | Yes ----------- 1  No ----------- 2  Don’t remember --------- 3  Don’t know --------- 4  No response ----------- 99  Other (specify)-------- 89 |
| 112 | If yes for Q 118 specify the method you /your  partner used? (More than one answer can be possible) | Abstained from sex --------- 1  Condom ----------- 2  Pill (OCP) ----------- 3  Injectable ------------ 4  IUD ---------- 5  Implants --------- 6  Tubal-ligation /Vasectomy ----7  No response ---------99  Other (specify) ------89 |
| 112 | Are you/your partner/ using Family planning method currently (during the study period)? | Yes ----------------- 1  No ---------------- 2  I don’t know --------------- 3  No response --------------- 99 |
| 113 | If yes for question 112, specify the method you  are using? (More than one answer can be possible) | Abstained from sex -------- 1  Condom ----------- 2  Pill(OCP) ----------- 3  Injectable ------------ 4  IUD ---------- 5  Implants --------- 6  Tubal-ligation /vasectomy ---7  No response ---------99  Other (specify) -----89 |
| 114 | (If the answer for Q112 No) would you like to use family planning method in the future? | Yes ----------- 1  No ------------ 2  Don’t know ---------- 3  No response --------- 99  Other (specify) ------- 89 |

| 115 | If yes for question 114, specify the method you intend to use? (More than one answer can be possible) | Abstained from sex --------- 1  Condom ----------- 2  Pill(OCP) ----------- 3  Injectables ------------ 4  IUD ---------- 5  Implants --------- 6  Tubal legation /vasectomy ---7  Other (specify) -------89 |
| --- | --- | --- |
| 116 | If the answers for question 114 no, why don’t you want to use family planning? | want to have a child -----1  fear that family planning drugs may create complication with ARV treatment -----2  I abstained from sex ----3  No response ----------99  Other specify------------89 |

**PART III – Information on reproductive characteristics**

| 117 | Have you had sexual Intercourse in the past six months? | Yes ---------------- 1  No --------------- 2  No response --------99  Other (specify) ------89 |
| --- | --- | --- |
| 118 | (If yes for Q 117) Have you used condom? | Yes -------------- 1  No ---------------- 2  I don’t remember ---3  No response ------ 99  Other (specify) -------89 |
| 119 | If yes for Q118 how often? | Always -------1  Some times ---------2  No response ---------99  Other (specify) --------89 |
| 120 | If the answer for question 118 yes, why do you used condom? | To prevent pregnancy ---- 1  Because my partner HIV status is negative 2  Health care providers advised me to use condom --- 3  No response --------99  Other (specify) -------89 |
| 121 | If the answer for question 118 no, why didn’t you used condom? | I want to have children -------1  My partner did not like it --- 2  No response --------99  Other (specify) ------89 |

**PART IV- Information on Child Desire**

| 122 | How many live births have you had in your life? | --------------Live births  I did not give birth at all ------97  I do not have any live birth ---98  No response ----- 99  Other (specify) -----89 |
| --- | --- | --- |
| 123 | How many live children do you have now? | No of alive children --------  I do not have children at all--- 97  I do not have alive children ----98  No response --------- 99  Other (specify) -------89 |
| 124 | Would you like to have children in the future? | Yes ------------- 1  No -------------- 2  Don’t know -------- 3  No response --------- 99  Other (specify) -------89 |
| 125 | If the answer for Q 124 yes, when do you prefer to have a child? | -----months /----------years  Don’t know---------98  No response --------- 99  Other (specify) -------89 |
| 126 | If the answer for Q 124 yes, How many  (more) children would you like to have in the  Future? | One-----------------96  Two and above---------- 97  Don’t know ----------- 98  No response ----------- 99  Other (specify) -------89 |
| 127 | (If the answer for question 124 is No) why do you not want to have children in the future? | have desired number of children -1  fear of mother to child HIV transmission risk ------ 2  don’t have adequate income to add  another child ------------ 3  Health care providers advise not to have a child ----------- 3  Child bearing may further compromise me/my partner health---------------4  No response -------- 99  Other (specify) -----------------89 |
| 128 | Does your husband /wife/ partner want to have a child in the future? | Yes ----------- 1  No ------------ 2  Don’t know ----------3  Don’t have partner ------- 4  No response ----------- 99  Other specify --------------89 |
